# Medico-legal assessment of physical abuses in asylum cases: a multidisciplinary role for multiform issues

**DOI:** 10.1007/s12024-025-01125-1

**Published:** 2025-11-07

**Authors:** Lorenzo Franceschetti, Francesca Magli, Lidia Maggioni, Stefano Tambuzzi, Jane Moffat, Danilo De Angelis, Cristina Cattaneo

**Affiliations:** 1 Forensic Laboratory of Anthropology and Odontology, Institute of Legal Medicine, Department of Biomedical Sciences for Health, LABANOF – Laboratorio di Antropologia e Odontologia Forense, University of Milan, via Luigi Mangiagalli 37, Milan, 20133 Italy; 2 Manchester University NHS Foundation Trust, Oxford Rd, Manchester, M13 9WL UK

**Keywords:** Clinical forensic medicine, Asylum seekers, Violence, Physical assessment, Multidisciplinary approach, Specialist examination, Forensic radiology, Istanbul protocol

## Abstract

With increasing migratory flows, forensic assessment of physical and psychological abuse plays an essential part for the proper functioning of humanitarian asylum procedures. Among professionals involved with vulnerable migrants, clinical forensic practitioners identify and assess injuries related to physical abuse and correlate them with the victims’ narrative. The present study assesses the importance of a multidisciplinary approach to the final assessment of scars, analysing its impact on the medico-legal evaluation of asylum seekers who suffered physical abuse. A retrospective study was conducted on all cases of asylum seekers evaluated at the Milan University Institute of Legal Medicine from 2008 to 2020 to investigate in which cases further investigations were needed and the outcome impacts of these investigations. Of the individuals examined, 92 asylum seekers were subjected to further forensic instrumental examinations (80.4%) and specialized medical consultations (33.7%). The most common indication for further investigation was for blunt shape forces in combination with other forces (38%), followed by blunt force injuries alone (34.8%). Radiography was the most widely used instrumental examination indicated to investigate injuries (90%) and the most frequent further consultation was odontological (17.4%). In 62 cases (47.7%) the presence of scars was confirmed by the identification of further skeletal and visceral lesions. The present research highlights the direct impact of a multidisciplinary, specialist approach on forensic consistency findings. This approach facilitated and improved the accuracy of clinical forensic evaluation in these highly sensitive cases, thereby reducing errors when assessing the presence of confounding factors, including those consequent from healing processes.

## Introduction

The increase in migratory flows for religious, political, racial, humanitarian and social reasons has increased exponentially in recent years, reaching epidemic proportions [1–5]. In this scenario, forensic assessment of physical violence either in the country of origin or en route is essential for the proper functioning of procedures for the recognition of political asylum and humanitarian protection [6–13].

In the literature, the studies carried out by Thomsen in 2000 [14] and by Sheather and collaborators in 2015 [15] emphasized the role of a thorough medico-legal examination to identify injuries related to physical violence, including torture. For this reason, multiple professional, special figures, dealing with vulnerable migrants, are necessary to respond to the specific medical, legal and social needs, particularly for assessment of asylum seekers’ physical and psychological condition. Health professionals, in addition to guidelines, need specialist tools and access to properly and thoroughly ascertain clinical conditions of asylum seekers [15–17]. Valuable guidelines for physical and psychological investigation and documentation of torture signs on asylum seekers are embodied by the Istanbul Protocol ([18]– [19]). The Manual, among other things, recommends that photography and instrumental (X-ray, CT, MRI, ultrasound) and specialist in-depth study (gynecologist, urologist, orthopedist, odontologist, etc.) should be a routine part of the medical examination as well as a formal investigation, after appropriate consent is acquired. Several scientific papers confirm the Istanbul Protocol efficiency and recommend implementing its applications [14, 20–22] and to incorporate it into the curricula of all medical experts who perform such delicate examinations ([23]– [24]).

To this end, the Municipality of Milan, one of the main cities in Northern Italy, approved – in 2012 through the Municipal Decree n.1674 – the creation of a network in the Milanese territory with the aim of assisting, supporting and caring for asylum seekers and especially vulnerable individuals. The Milan University Institute of Legal Medicine is one of team’s key member and an active collaborator, performing clinical forensic evaluation of scars and/or lesions in those asylum applicants who have declared to be a victim of physical abuse, violence or torture, in order to determine the compatibility between narrative of physical violence and physical evidence of available.

This study was aimed at focusing on evaluating the role and impact of a multidisciplinary approach on the final assessment of scars and/or lesions in the medico-legal evaluation on asylum seekers who have suffered from physical violence. The purpose was to discuss the experience in clinical forensic medicine from the University Institute of Legal Medicine in Milan and the attempt of the Italian Ministry of Health to ameliorate the present situation through the introduction of a decree, on April 3rd, 2017, which aims to protect asylum seekers, in particular those in vulnerable conditions, during the process of recognition of international protection, creating the conditions so that victims of traumatic events can be adequately protected [25].

## Materials and methods

Overall, 290 asylum seekers underwent physical examination at the University Institute of Legal Medicine over a decade (2008–2020). These individuals were referred by the Municipality of Milan in order to perform a complete medico-legal evaluation on reported lesions. During the clinical assessment, a full medical examination of the alleged victims, according to the Istanbul Protocol, was performed. A final report was drafted on the consistency between the allegations of physical violence and the external examination evaluations, then submitted to the Territorial Commission. All individuals were examined by forensic specialists and anthropologists, always with the presence of a mediator, a translator, or a trusted person. The interview also concerned the reasons why they fled their countries, the events of violence with details concerning the physically traumatic events.

To thoroughly investigate the reported lesions, for 92 individuals a multidisciplinary specialistic approach was indicated: in detail, forensic instrumental examinations, such as radiography, CT, MRI, as well as specialized medical consultations.

The study evaluated the number of additional examinations, the specific type of evaluations requested and the impact of impact of the specialist referral or examination on the physical assessment of violence and the consistency findings between the individual’s allegation and the physical evidence.

Sex and age were not taken into consideration since the specific population was mostly composed of young adult males, on average between twenty and thirty years of age. The limited number of women in our case records prevented a meaningful comparative analysis between males and females (for specific data cfr. ref. 12,24). However, both sexes were included in the study population for the purposes of analyzing specialist examinations.

Data was obtained from these medical records which were recorded and entered into a digital data set and subsequently extrapolated and analyzed with descriptive statistics using Excel^®^ software. In all cases, details have been anonymized. The results were analyzed and then grouped into frequency tables, expressed as percentages. Institutional Review Board approval for the study was obtained, as the patients provided informed consent for the specific release and use of their data for research purposes.

## Results

Of the ninety-two individuals, 74 (80.4%) were referred for, and underwent, forensic instrumental examinations. Sixteen (17.4%) were referred for to dental examination by a forensic odontologist and 15 (16.3%) for specialized medical consultation, including gynaecological, ophthalmological, urological, neurological, orthopaedic, and surgical assessments. In some cases, both radiological examinations and medical consultations were performed.

**Fig. 1:**
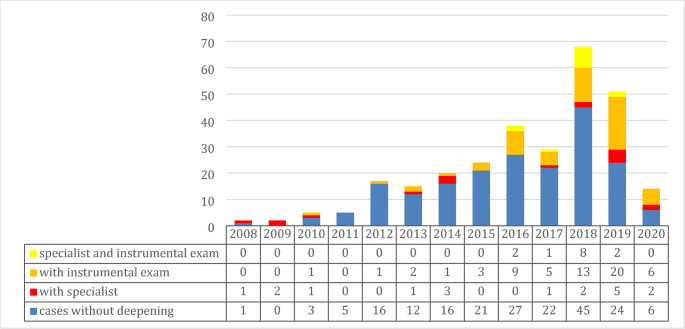
Annual distribution of asylum seekers assessed at the University Institute of Legal Medicine (Milan) from 2008 to 2020, according to the depth of forensic investigation. Bars show the number of cases without further investigation (blue), with specialist consultation only (red), with instrumental examination only (orange), and with both specialist and instrumental examinations (yellow). Data are presented as absolute numbers per year

Forensic instrumental examinations included radiographs (*n* = 117, 90.0%), CT scan (*n* = 7, 5.4%), MRI (*n* = 5, 3.8%) and ultrasonography in one case (0.8%). In particular, 17 (13.1%) instrumental examinations of the head, 12 (9.2%) of the chest, 47 (36.2%) of upper limbs, 52 (40%) of lower limbs and 2 (1.6%) of the entire skeleton. In multiple patients, more than one body district was radiologically assessed, according to the reported account and the injuries suffered. Overall, the mean was 1.4 instrumental examinations per person (Fig. 1; Table 1).

**Table 1:** Annual distribution of asylum seekers assessed at the university Institute of legal medicine (Milan) from 2008 to 2020. The table reports the number of subjects who underwent forensic evaluation, odontologist examination, specialist consultation, and instrumental examination. Some individuals received more than one type of assessment

	Subjects with forensic evaluations	Subjects with odontologist exam	Subjects with specialistic examination	Subjects with instrumental examination
2008	2	1	0	0
2009	2	2	0	0
2010	5	1	0	1
2011	5	0	0	0
2012	17	0	0	1
2013	15	1	0	2
2014	20	2	1	1
2015	24	0	0	3
2016	38	0	2	11
2017	29	0	2	6
2018	68	5	5	21
2019	51	4	3	22
2020	14	0	2	6
**TOTAL**	**290**	**16**	**15**	**74**

**Fig. 2:**
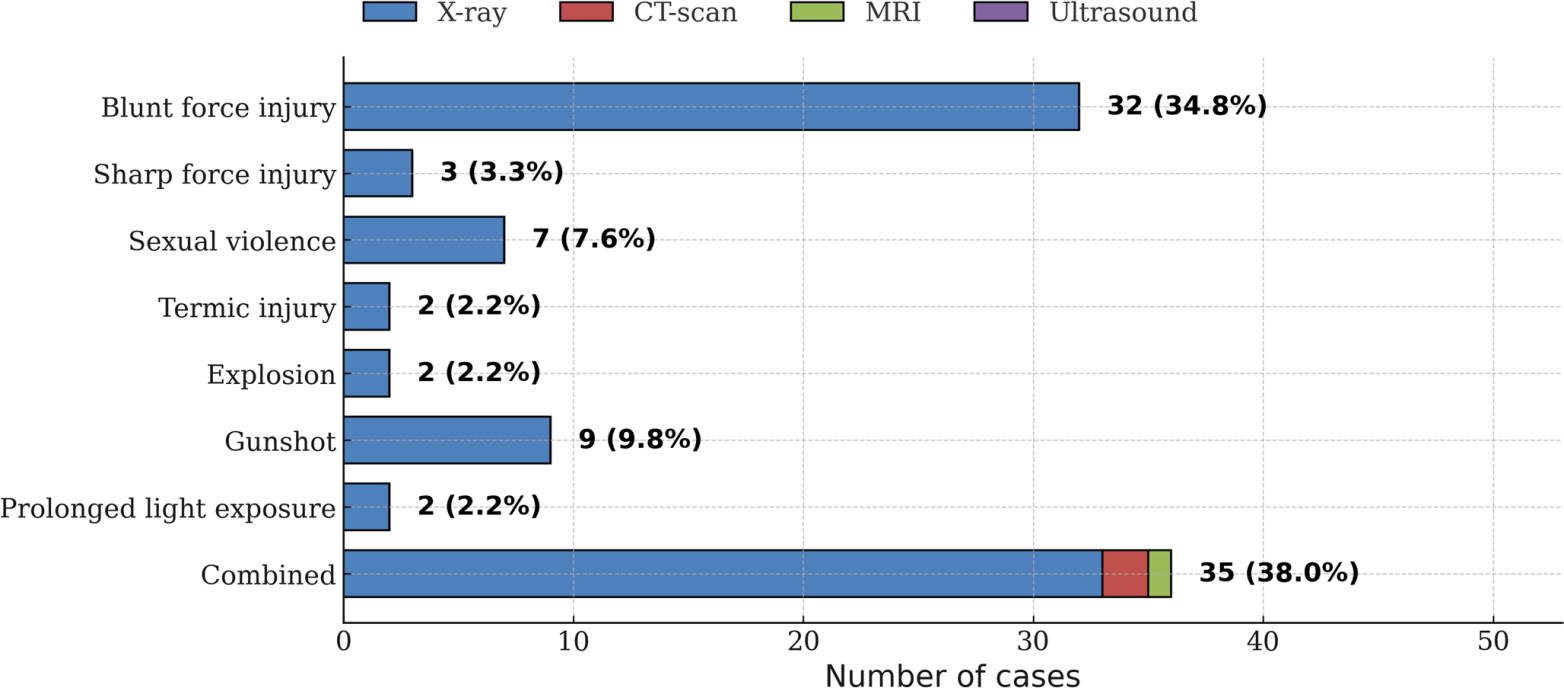
Distribution of injury types among asylum seekers assessed at the University Institute of Legal Medicine (Milan), 2008–2020. Each bar represents the number and percentage of cases for each type of injury, with further breakdown by the type of instrumental examination performed (X-ray, CT-scan, MRI, ultrasound). “Combined” refers to cases with multiple mechanisms of injury. Data labels within the bars indicate the absolute number and percentage of cases for each category

The most commonly investigated injury type was the combined blunt shape force accompanied by other types (*n* = 35, 38.0%), followed by the blunt force injury alone (*n* = 32, 34.8%), gunshot wounds (*n* = 9, 9.8%), sexual violence (*n* = 7, 7.6%), sharp force injury (*n* = 3, 3.3%), thermal lesions (*n* = 2, 2.2%), explosion related wounds (*n* = 2, 2.2%) and prolonged forced exposure to light (*n* = 2, 2.2%) (Fig. 2).

**Fig. 3:**
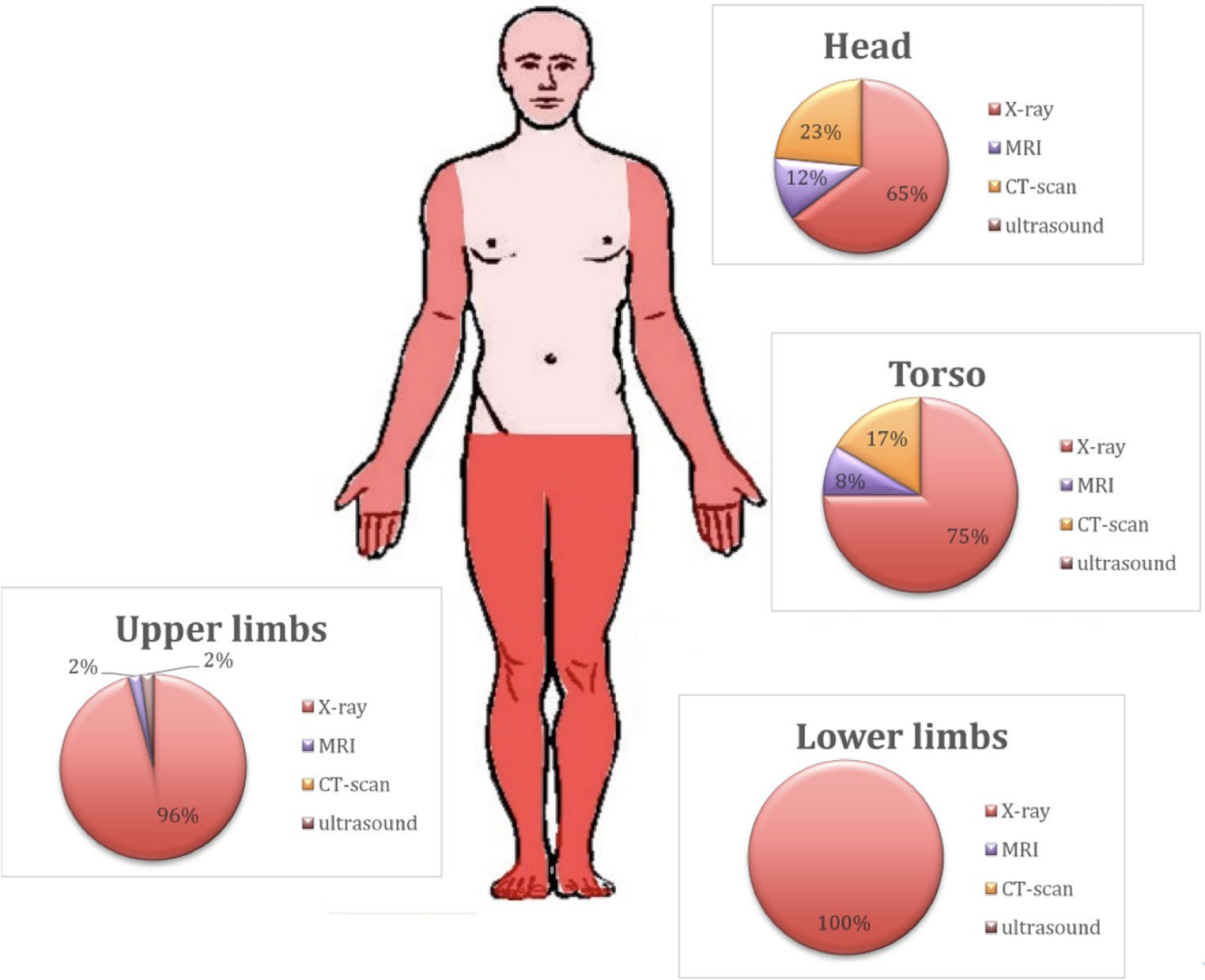
Distribution of instrumental examinations by anatomical region among asylum seekers assessed at the University Institute of Legal Medicine (Milan), 2008–2020. The central diagram highlights in red the body regions most frequently investigated. The pie charts show the relative proportion of X-ray, CT-scan, MRI, and ultrasound examinations performed for the head, torso, upper limbs, and lower limbs, respectively. Data are presented as percentages for each modality within each anatomical region

Radiography was the most widely used instrumental examination to investigate injuries, especially in the context of the blunt force injury, which was the key type of injury where the mode of instrumental examinations (MRI, CT, X-ray, ultrasound) deepened the capacity for visualization of the lesion (Fig. 3).

Dental examinations were performed in cases where subjects reported having suffered trauma to the mouth, with some cases reporting broken teeth. Dental examinations were also typically associated with a radiograph.

The other specialized medical consultations were also carried oud when helpful according to the type of violence, abuse or torture suffered and to investigate specific symptoms reported. In detail, gynecological examinations were requested in 4 cases, orthopedic and neurological examinations in 3 cases each, urological and ophthalmological examinations in 2 cases each, and finally, surgical examination in 1 case.

**Fig. 4:**
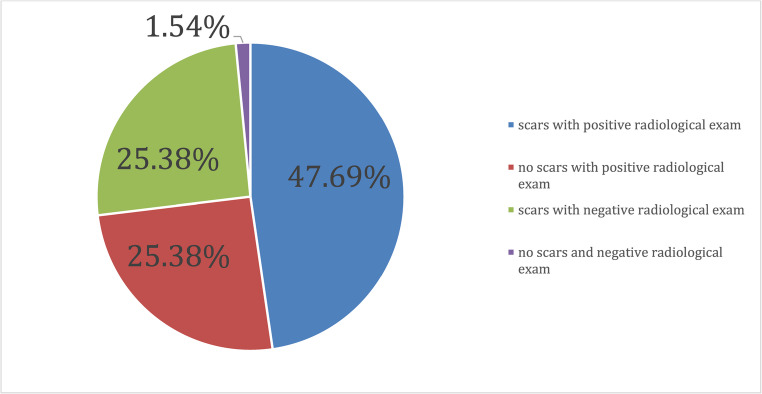
Distribution of cases by the concordance between clinical scars and radiological findings in asylum seekers assessed at the University Institute of Legal Medicine (Milan), 2008–2020. The pie chart shows the proportion of cases with scars and positive radiological exam, no scars with positive radiological exam, scars with negative radiological exam, and no scars with negative radiological exam

For each case, reports of radiological examinations and/or specialized medical consultations were acquired and carefully assessed (Fig. 4).

Of the examined cases, 47.7% (*n* = 62) showed both clinically appreciable scars and positive radiological findings, confirming the consistency between self-reported traumatic events and objective evidence. Notably, in 25.4% (*n* = 33) of cases, radiological imaging identified underlying skeletal or visceral lesions despite the absence of visible scars. This finding underscores the crucial medico-legal role of instrumental examinations, especially in victims of blunt trauma or torture techniques designed to minimize external marks (e.g., falanga, suspension). Conversely, 25.4% (*n* = 33) of cases presented clinical scars but no radiological evidence of underlying injuries, a scenario possibly attributable to superficial soft tissue trauma, healing dynamics, or technical limitations of imaging. Only a minority (1.5%, *n* = 2) showed neither scars nor radiological lesions, raising the need for a nuanced medico-legal interpretation in light of the patient’s narrative, the time elapsed since the alleged event, and the inherent limitations of post-trauma forensic assessment.

Finally, in all cases, the reports of specialized medical consultations proved to be crucial for the proper assessment according to the Istanbul Protocol. In detail, gynecological examinations made it possible to search for and detect signs of previous sexual violence (e.g. genital mutilations), while orthopedic, surgical, neurological and urological examinations made it possible to better assess the outcome of blunt and sharp trauma to joints and internal organs, depending on the body districts involved. Ophthalmologist examinations were essential in cases of reported prolonged forced exposure to light.

## Discussion

### Context

Asylum applicants seeking international protection on the basis of torture were examined at the University Institute of Legal Medicine in Milan. These evaluations occurred at various time intervals following the reported traumatic events, in some cases up to three years later [26–28]. This delay often posed significant challenges for medical and forensic assessments, especially in identifying and characterising physical evidence of torture.

### Nature of injuries and healing processes

In the present study, asylum seekers undergoing medical evaluations in the City of Milan most frequently reported blunt force as the primary mechanism of injury. This category encompassed various forms of assault, including beating, kicking, punching, and the use of blunt objects and ligatures. Less frequently reported were injuries caused by sharp force, thermal agents, gunshot wounds, explosions, and electrocution. Regarding sexual abuse physical evaluation shows lesions in low percentages, because the most frequent consequence of sexual violence is psychological [8, 29–31].

Physical violence typically resulted in abrasions, bruises, lacerations, bleeding, or fractures. Healing processes usually led to either complete recovery, known as *restitutio ad integrum*, or to the formation of scars. Occasionally, the healing process produced atypical scars, especially in individuals with darkly pigmented skin. In such cases, hypertrophic or abnormal scarring, including keloids, were observed [32–35]. Conversely, in some instances, healing left no externally visible lesions. These latter cases were particularly problematic for forensic assessments because survivors who reported having experienced physical violence but showed no physical signs, were difficult to evaluate from a medico-legal perspective. This difficulty was especially pronounced in cases involving blunt force trauma, where the lack of detectable scars made it challenging to correlate the individual’s narrative with the alleged violence, as visible signs may have disappeared during the healing process.

Furthermore, in many countries along migration routes, specific methods of torture are used deliberately to avoid leaving external marks. These methods include various forms of body suspension or foot whipping. As a result, external physical examinations may fail to detect signs of torture, making them not only insufficient but potentially counterproductive.

### Limitations in current assessment practices

Few studies have comprehensively analysed the characteristics of injuries and healing processes in torture victims [34, 36–40]. Notable examples include Perera’s study on torture scars in Sri Lanka and Arnold’s work on wound and scar management. Although the current literature provides examples of scar assessment methods [20, 23, 37, 41–46], the medico-legal evaluation of physical violence still presents significant limitations.

Despite the importance of specialist assessments, the involvement of forensic pathologists remains limited. Victims are often examined by general practitioners or non-forensic personnel, or in many cases, not examined at all [42]. This gap highlights the urgent need for changes in healthcare and judicial practice. Governments and healthcare systems should begin implementing enhanced assessment measures through multidisciplinary interventions. Proper identification, documentation, and treatment of victims of physical violence, including torture, is a responsibility that every country must strive to uphold [14, 47–49].

### National and international guidelines

In line with this perspective, the Italian Ministry of Health issued guidelines on 3 April 2017, focusing on the assistance, rehabilitation, and psychiatric care of refugees and asylum seekers who have been subjected to torture, rape, or other severe forms of physical, psychological, or sexual violence [25]. According to these guidelines, applicants must be granted access to proper medico-legal assessment and certification in accordance with the Istanbul Protocol, as well as access to the national healthcare system.

Victims of violence are entitled to rehabilitation in every case, and healthcare professionals are obliged to apply all available resources to ensure appropriate standards of care. These resources include instrumental investigations and specialist consultations. Although the guidelines are significant, their practical application remains limited. In most cases, the only viable pathway to care is through Emergency Departments. Even then, access to specialist consultations within the National Health Service is often hindered by the lack of proper documentation. While the Istanbul Protocol recommends specialist evaluations, there are considerable practical difficulties in implementing this recommendation in Italy.

### The role of multidisciplinary and instrumental evaluation

The accurate assessment of torture victims necessitates more frequent use of instrumental diagnostics and specialist consultations. In forensic practice, a multidisciplinary approach should become standard when evaluating asylum seekers. For example, the use of X-ray examinations has increased due to their ability to quickly assess various parts of the body. In this study, the most frequently radiologically examined areas were the limbs, particularly the hands and forearms, where defensive injuries are common. These X-rays revealed the presence of bone calluses or retained metal fragments from past trauma or post-traumatic surgical procedures.

The integration of forensic and instrumental evaluations proved beneficial in determining the origins of scars and in corroborating asylum seekers’ narratives. Blunt force trauma, given its high frequency, variability of wounding instruments, and diversity of scar formation, was the most commonly investigated type of injury. This was followed by injuries resulting from gunshots, sharp objects, and explosions.

These findings reinforce earlier observations made by Clément et al. in 2017, who emphasized the need for more detailed examinations of torture victims using instrumental methods [46]. The involvement of other professionals allowed for a more comprehensive approach to each case. The forensic pathologist plays a pivotal role in determining which specialist examinations are appropriate based on the survivor’s account and the areas of the body affected.

For instance, in cases of sexual violence, a gynaecological examination is essential for women, while a urological or surgical consultation may be necessary for men. In instances of blunt or penetrating trauma, orthopaedic, neurological, or surgical evaluations may be warranted, especially if joint or visceral injuries are suspected. These assessments can corroborate the victim’s account even in the absence of visible scarring. In their absence, forensic assessments may be incorrect or incomplete.

There are specific forms of torture, such as suspension, that may not result in broken bones but can cause nerve and muscle injuries due to joint strain. These injuries are more easily identified through targeted specialist evaluations. Similar considerations apply to foot whipping, sensory impairments, and nerve damage. Ultimately, this detailed evaluation process enables a more accurate classification of injuries, identification of the harmful instruments used, and a more precise judgement of consistency between clinical findings and the survivor’s narrative.

It is worth noting that in approximately a quarter of the evaluated cases, no external scars were present, yet skeletal or visceral lesions were identified through radiographic or other instrumental methods. Nevertheless, instrumental and clinical assessments may still yield no objective findings. As such, while routine use of instrumental investigations may not resolve every individual case, they significantly contribute to a more nuanced understanding of the medico-legal role in evaluating allegations of torture.

### Timing and quality of evaluations

The timing of medico-legal evaluations is another crucial factor. Clinical forensic literature has consistently highlighted the importance of timely assessments to properly document injuries. However, in practice, these evaluations often take place around one year or more after the traumatic event, complicating assessments due to the natural healing process and changes in scar appearance (26–28, 41).

### Flow of information and final considerations

Establishing a structured flow of information between forensic teams and asylum review committees is essential. This exchange, which should include instrumental findings and specialist insights, could substantially aid in recognising human rights violations. It would also enhance the preparedness and effectiveness of professionals involved in the process.

This study highlights how the multidisciplinary approach adopted in these cases significantly improved clinical forensic evaluations and reduced the likelihood of errors, particularly when assessing confounding factors related to the healing process. Although the necessity of an extensive and interdisciplinary approach is widely acknowledged, its practical implementation remains insufficient. For accurate evaluation of each individual case, collaboration among various professionals is imperative. Training and institutional support are crucial in preparing these professionals for the complex task of evaluating torture survivors. Looking forward, a broader integration of specialized support within clinical forensic medicine is both expected and necessary to provide forensic practitioners with effective tools for assessing physical violence.

## Limitations

This study presents several limitations. First, the demographic profile of the sample predominantly includes young adult males, limiting the generalizability of findings to other age groups or female subjects. This gender imbalance constrains comparative analyses that could steer to a more nuanced understanding of injury patterns across different gender and age demographics.

Another limitation concerns the use of radiographic and instrumental examinations, which were performed selectively based on clinical indications rather than uniformly across all cases. This approach, while guided by specific injury types, may have resulted in differential detection rates, potentially underrepresenting some types of trauma, especially those not routinely subjected to imaging.

The study’s focus on physical injuries means that it does not comprehensively address psychological trauma, which is often a significant component of torture and violence cases. As such, the absence of mental health assessments leaves a gap in understanding the full extent of harm experienced by the subjects.

Finally, the lack of a control group of non-asylum seekers limits the ability to distinguish findings specific to this population, which would be essential for isolating indicators directly related to the traumatic experiences typically associated with migration and persecution contexts.

## Conclusions

This study highlights the critical importance of a multidisciplinary approach in the forensic assessment of asylum seekers who report physical violence, particularly in cases of torture. Integrating instrumental examinations with specialized consultations enables a more comprehensive analysis of injuries, uncovering evidence often missed by external examinations alone. This approach not only enhances the accuracy of medico-legal evaluations, but also strengthens the accuracy of reports submitted to Committees assessing asylum applications. Consistent application of detailed assessments, according to the Istanbul Protocol standards, is essential for substantiating claims of human rights violations and maximising the use of available national legal instruments. Future developments in forensic clinical practice should aim to standardize and expand access to these multidisciplinary resources, ensuring that practitioners are equipped to handle the complexity of cases involving alleged torture and physical violence. Such advancements could serve as foundational steps toward a more effective and humane asylum assessment process.

## Key points

1. Multidisciplinary approach improves forensic evaluation of asylum seekers.
2. Instrumental exams reveal hidden injuries in alleged torture cases.
3. Specialist consultations confirm and detail lesion patterns.
4. Adopting Istanbul Protocol enhances medico-legal reporting accuracy.

## Data Availability

Data are available on reasonable request.
